# Effects of ethanol and ethanol metabolites on intrinsic function of mesenteric resistance arteries

**DOI:** 10.1371/journal.pone.0214336

**Published:** 2019-03-20

**Authors:** Jonathan M. Eby, Matthias Majetschak

**Affiliations:** 1 Department of Surgery, Loyola University Chicago Stritch School of Medicine, Maywood, Illinois, United States of America; 2 Alcohol Research Program (ARP), Loyola University Chicago Stritch School of Medicine, Maywood, Illinois, United States of America; 3 Department of Surgery, Morsani College of Medicine, University of South Florida, Tampa, Florida, United States of America; 4 Department of Molecular Pharmacology and Physiology, Morsani College of Medicine, University of South Florida, Tampa, Florida, United States of America; Medical College of Georgia, Augusta, UNITED STATES

## Abstract

Evidence suggests that ethanol-induced hypertension is associated with increased cardiovascular responsiveness to vasopressors in vivo and enhanced reactivity of isolated arteries to vasopressors ex vivo. The underlying mechanisms are not well understood and the contribution of ethanol metabolites to vascular effects induced by ethanol consumption are unclear. Mesenteric resistance arteries were harvested from Sprague-Dawley rats. Pressure myography was utilized to test effects of ethanol, acetaldehyde and phosphatidylethanol on myogenic tone and on vasoconstriction induced by phenylephrine, arginine vasopressin (aVP), endothelin-1 and KCl. Ethanol, acetaldehyde and phosphatidylethanol concentrations were monitored during the experiments. Ethanol concentrations in the vessel bath decreased with a half-life of 25min; acetaldehyde and phosphatidylethanol concentrations remained constant. Pretreatment with ethanol dose-dependently increased the potency of phenylephrine to induce vasoconstriction 4-*fold* (p<0.01). These effects were comparable when arteries were pre-treated with a single dose of ethanol for 30min and when ethanol concentrations were kept constant during 30min and 60min of pretreatment. While ethanol also dose-dependently increased the potency of aVP to induce vasoconstriction 1.7-*fold* (p<0.05), it did not affect vasoconstriction induced by endothelin-1 or KCl. Acetaldehyde pre-treatment (30 min) dose-dependently increased the potency of phenylephrine to induce vasoconstriction 2.7-*fold* (p<0.01) but did not affect other vasoconstrictor responses. Phosphatidylethanol did not affect any vasoconstrictor responses. Ethanol and its metabolites did not affect myogenic tone. These data suggest that ethanol and acetaldehyde selectively sensitize intrinsic constrictor responses upon activation of vascular α_1_-adrenergic and/or vasopressin receptors at clinically relevant concentrations. Our findings support the concept that enhanced vasoreactivity to vasoactive hormones contributes to the development of hypertension induced by ethanol consumption. Ex vivo exposure of resistance arteries to ethanol and acetaldehyde resembles effects of chronic ethanol consumption on intrinsic vascular function, and thus could serve as test platform to evaluate interventions aimed to mitigate vascular effects associated with ethanol consumption.

## Introduction

Cardiovascular diseases are major health problems world-wide, affecting over 85 million people in the US alone [1]. During the past century, associations between chronic ethanol consumption and various cardiovascular diseases, such as arterial hypertension, stroke or myocardial infarction, have been firmly established [1, 2]. The effects of alcohol consumption on the cardiovascular system are complex and appear to depend on the dose and mode of alcohol consumption [3, 4]. While epidemiological studies suggested a U-or J-shaped dose-effect relationship between alcohol consumption and cardiovascular disease, with low-to-moderate drinking being beneficial, more recent epidemiological methodologies question this association [4]. It is well accepted, however, that chronic moderate-to-heavy drinking has detrimental effects on cardiovascular health [2, 4]. Chronic moderate-to-heavy drinking induces arterial hypertension in humans and animals [2–14]. This association is of major importance as hypertension is the single most important risk factor for global burden of disease and significant percentages of hypertension can be attributed to ethanol consumption [2, 15, 16].

The effects of ethanol consumption on blood pressure regulation are thought to involve the central and autonomic nervous systems [17, 18]. Furthermore, several lines of evidence suggest that ethanol has direct effects on vascular function and that ethanol-induced hypertension is associated with increased cardiovascular responsiveness to vasopressors *in vivo* and enhanced reactivity of isolated arteries to vasopressors *ex vivo* [2, 6, 7, 12, 17–20]. The mechanisms by which ethanol consumption alters intrinsic vascular function, however, are not well understood. Furthermore, the contribution of ethanol metabolites to alterations in intrinsic vascular function induced by ethanol consumption are unclear. As multiple previous reports suggested that vasoconstrictor responses upon α_1_-adrenergic receptor (AR) activation are sensitized after chronic alcohol consumption [2, 6, 7, 21, 22], we tested whether such effects are also detectable in resistance arteries upon ethanol exposure ex vivo. We observed that a short exposure of isolated mesenteric resistance arteries to clinically relevant concentrations of ethanol sensitizes intrinsic vasoconstrictor responses to the selective α_1_-adrenergic receptor agonist phenylephrine (PE), thus providing a simple test platform that recapitulates vascular consequences of ethanol consumption. Utilizing this test platform for detailed dose-response experiments with ethanol and its metabolites acetaldehyde and phosphatidylethanol, we provide evidence that ethanol and acetaldehyde have direct effects on vascular function, and dose-dependently and selectively sensitize intrinsic vasoconstrictor responses of mesenteric resistance arteries upon activation of α_1_-adrenergic- and/or arginine vasopressin receptors. Our findings provide new insights into pathophysiological consequences of ethanol and ethanol metabolite exposure on the vasculature, which are likely to contribute to the etiology of hypertension induced by ethanol consumption.

## Materials and methods

### Proteins and reagents

Ethanol, acetaldehyde, phenylephrine, arginine vasopressin, endothelin-1 and KCl were purchased from SigmaAldrich (St. Louis, MO). Phosphatidylethanol was purchased from Avanti Polar Lipids, Inc (Alabaster, AL).

### Pressure myography

Pressure myography was performed as described in detail previously, with slight modifications [23–27]. Male Sprague-Dawley rats weighing 300-350g were obtained from Envigo (Huntingdon, United Kingdom). All procedures were performed according to National Institutes of Health Guidelines for Use of Laboratory Animals and approved by the Loyola University Chicago Institutional Animal Care and Use Committee. Rats were anesthetized with 3.5% isoflurane, the mesentery was excised and animals were euthanized by bilateral pneumothorax and cardiectomy. The mesentery was immediately placed in a solution containing 130 mM NaCl, 4.7 mM KCl, 1.18 mM KH_2_PO_4_, 1.17 mM MgSO_4_, 14.9 mM, NaHCO_3_, 5.5 mM D-Glucose, 0.026 mM EDTA, 1.16 mM CaCl_2_ aerated with 95% O_2_, 5% CO_2_ at 37°C (PSS). Third or 4^th^ order mesenteric resistance arteries were dissected free from adipose and connective tissue prior to mounting onto two glass cannulae with United States Pharmacopeia (USP) scale 11–0 sutures (Rockville, MD). Vessels were perfused and superfused with PSS, then pressured to 80 mmHg in a DMT 110P pressure myography system (DMT-USA, Ann Arbor, MI) for 5 minutes prior to starting each experiment. The vessel bath superfusion was continuously aerated with 95% O_2_, 5% CO_2_ throughout the experiment. Vessels were pretreated with ethanol or one of its metabolites for 30 minutes prior to the addition of increasing doses of PE, arginine vasopressin (aVP), endothelin-1, and KCl to the vessel bath. Changes of the outer diameter (o.d.) of the pressurized vessel were measured continuously throughout the experiment via digital video-edge detection.

To study the effects of ethanol and its metabolites on myogenic tone, arteries were pressurized to 80 mmHg and pre-incubated with ethanol and its metabolites for 30 min. The intraluminal pressure was then reduced to 0 mmHg and increased by 20 mmHg every 5 min. Changes in the outer diameter of the arteries were measured as described above.

### Measurements of ethanol concentrations

Ethanol concentrations in the vessel bath were measured using an AM1 Alcohol Analyzer (Analox, Huntington Beach, CA) according to the manufacturer’s instructions.

### Acetaldehyde assay

Acetaldehyde concentrations in the vessel bath were measured using the colorimetric EnzyChrom Acetaldehyde Assay kit (BioAssay Systems, Hayward, CA), which is based on aldehyde dehydrogenase catalyzed oxidation of acetaldehyde, in which the formed NADH reduces a formazan reagent. The intensity of the product color, measured at 565 nm, is directly proportional to the acetaldehyde concentration in the sample. Assays were performed as per manufacturer’s protocol.

### Phosphatidylethanol enzyme linked immunosorbent assay

Phosphatidylethanol concentrations in the vessel bath were measured with a phosphatidylethanol ELISA (Echelon Biosciences, Salt Lake City, UT) according to the manufacturer’s protocol.

### Data analyses

Data are expressed as mean ± standard error of the mean (SEM) from n independent experiments. Experiments were performed on different days using different animals. Data analyses were performed using the GraphPad-Prism 7 software. Data were analyzed by one-way analyses of variance (ANOVA) with Dunnett’s multiple comparison post-hoc test. Dose response curves and half-life decay curves were analyzed using non-linear regression analyses. A two-tailed p < 0.05 was considered significant.

## Results

**Fig 1:**
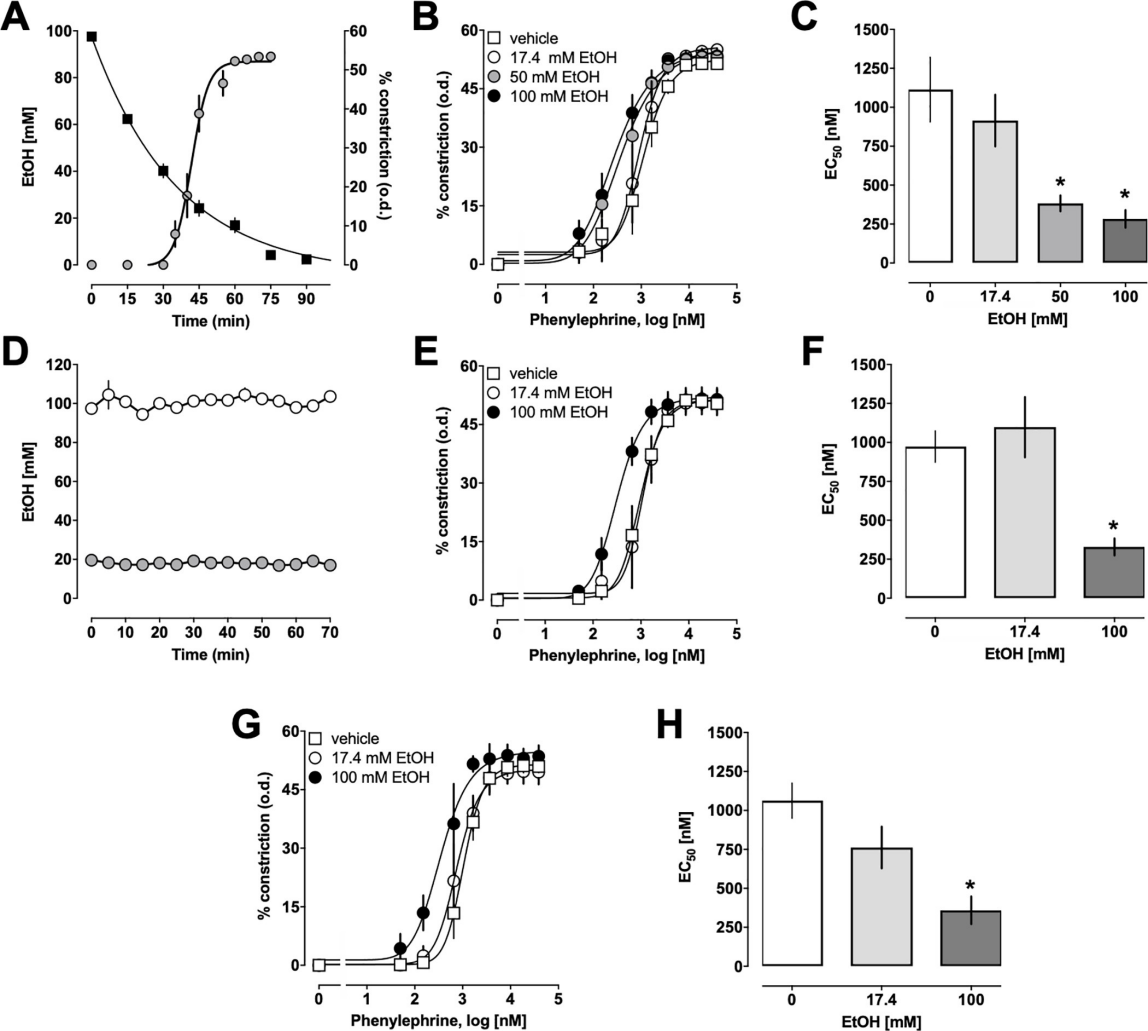
Effects of ethanol (EtOH) on phenylephrine-induced constriction of mesenteric resistance arteries. Data are mean ± SEM. **A.** EtOH concentrations in the vessel bath during pressure myography experiments. Arteries were pressurized to 80 mmHg, pretreated for 30 min with a single dose of 100 mM EtOH and exposed to increasing doses of phenylephrine (PE). Squares: EtOH concentrations (mM) in the vessel bath, n = 3–5. Circles: Vasoconstriction in % of the outer diameter (o.d.) in response to increasing doses of PE (n = 5). **B.** PE-induced constriction in % of the o.d. in arteries pretreated with single doses of EtOH for 30 min, as in A. Vehicle—n = 5; 17.4 mM EtOH–n = 3; 50 mM EtOH–n = 5; 100 mM EtOH–n = 5. **C.** EC_50_ concentrations for the vasoconstrictor effects of PE (mM) from dose-response curves in B. *: p<0.05 vs. vehicle. **D.** Ethanol concentrations (open circles: 100 mM EtOH; grey circles: 17.4 mM EtOH) were measured in 5 min intervals and maintained constant by adding ethanol to the vessel bath to account for evaporative losses. N = 3. **E.** PE-induced constriction in % of the o.d. in arteries pretreated with constant concentrations of EtOH for 30 min. Vehicle–n = 5; 17.4 mM EtOH–n = 3; 100 mM EtOH–n = 4. **F.** EC_50_ concentrations for the vasoconstrictor effects of PE (mM) from dose-response curves in E. *: p<0.05 vs. vehicle. **G.** PE-induced constriction in % of the o.d. in arteries pretreated with constant concentrations of EtOH for 60 min. Vehicle–n = 3; 17.4 mM EtOH–n = 3; 100 mM EtOH–n = 3. **H.** EC_50_ concentrations for the vasoconstrictor effects of PE (mM) from dose-response curves in G. *: p<0.05 vs. vehicle.

To test for direct effects of ethanol on α_1_-AR-mediated vasoconstriction, mesenteric resistance arteries were pre-incubated with ethanol for 30 min, followed by stimulation with increasing doses of the selective α_1_-AR agonist PE. Because ethanol evaporates from the aerated vessel bath during the experiment, we monitored ethanol concentrations in 15 min intervals. Fig 1A shows the time course of ethanol concentrations in the vessel bath along with the vasoconstrictor effects of PE when ethanol was added at a starting concentration of 100 mM. Ethanol concentrations in the vessel bath decreased with a half-life of 24.86 min (95% confidence interval: 21.5–29.5 min, n = 3 (t = 0–90 min)– 8 (t = 0–60 min)) under our experimental conditions. Addition of ethanol alone to the vessel bath did not induce vasoconstriction. Fig 1B shows the PE-induced vasoconstrictor effects when mesenteric resistance arteries were pre-incubated in the vessel bath with various concentrations of ethanol. While a starting concentration of 17.4 mM ethanol in the vessel bath, which reflects the legal blood alcohol limit for the operation of a vehicle in the US, did not alter the vasoconstrictor responses to PE, pre-incubation of mesenteric resistance arteries with a starting vessel bath concentration of 50 mM and 100 mM ethanol resulted in a left-shift of the vasoconstrictor responses to PE (Fig 1B). While ethanol pre-exposure of the arteries did not significantly affect maximal constriction induced by PE or Hill-slopes of the dose-response curves, ethanol dose-dependently increased the potency of PE to induce vasoconstriction ~ 4-*fold* (Fig 1C; EC_50_ (nM, mean ± SEM): 0 mM ethanol (vehicle) - 1114 ± 207; 17.4 mM ethanol 915 ± 166; 50 mM ethanol– 383 ± 51 (p = 0.003 vs. vehicle); 100 mM ethanol– 283 ± 56 (p = 0.001 vs. vehicle)). We then tested the effects of ethanol when ethanol concentrations were maintained constant during the experiment. As before, arteries were pre-exposed to ethanol for 30 min, followed by stimulation with PE. Ethanol concentrations were measured in 5 min intervals and maintained constant by adding ethanol to the vessel bath to account for evaporative losses (Fig 1D). The vasoconstrictor dose-responses to PE in arteries exposed to a constant concentration of 17.4 mM ethanol were indistinguishable from those in arteries not exposed to ethanol. In contrast, exposure to a constant concentration of 100 mM ethanol resulted in a left-shift of the dose response curves to PE (Fig 1E). The EC_50_ concentration for the vasoconstrictor effects of PE in arteries pre-exposed to a constant concentration of 100 mM of ethanol was significantly reduced to 329 ± 54 nM, as compared with arteries not exposed to ethanol (Fig 1F; vehicle: 973 ± 101 nM, p = 0.0057). To assess whether a longer pre-exposure would affect the sensitizing effects of ethanol, we then extended the pre-incubation period to a constant ethanol concentration to 1 hour (Fig 1G and 1H). This, however, did not affect the dose-dependency of the sensitizing effects of ethanol on PE-induced vasoconstriction (EC_50_ (nM, mean ± SEM)): vehicle– 1064 ± 113; 17.4 nM ethanol– 762 ± 134; 100 nM ethanol– 359 ± 88 (p = 0.0082 vs. vehicle)). Pre-exposure of arteries to constant ethanol concentrations for 30 min and 60 min did not significantly affect maximal constriction or Hill-slopes of the dose-response curves for PE. Furthermore, the EC_50_ concentrations for the vasopressor effects of PE in arteries pre-exposed to evaporating concentrations of ethanol for 30 min, to constant concentrations of ethanol for 30 min and to constant concentrations of ethanol for 60 min were not significantly different for each ethanol concentration.

**Fig 2:**
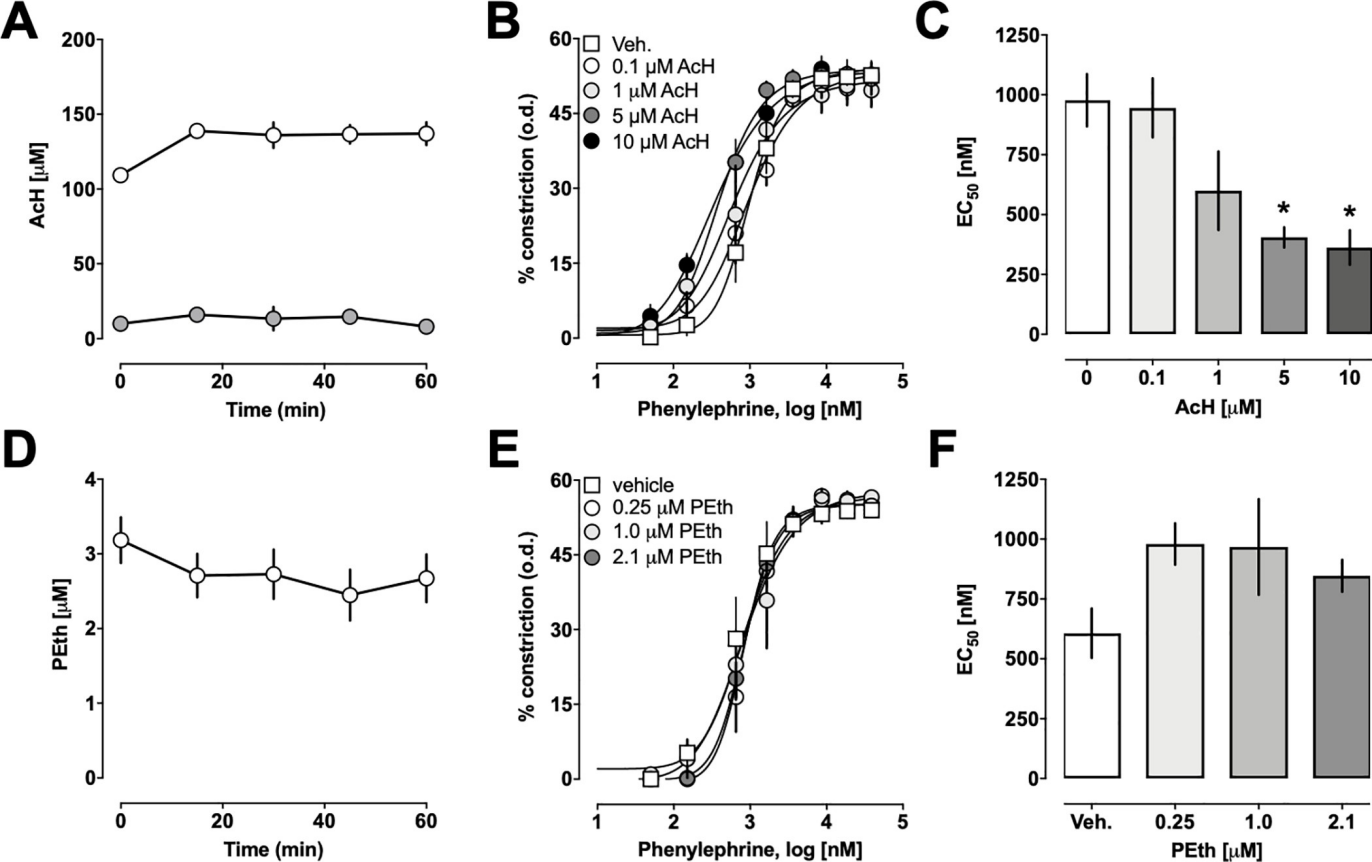
Effects of acetaldehyde (AcH) and phosphatidylethanol (PEth) on phenylephrine-induced constriction of mesenteric resistance arteries. Pressure myography experiments with arteries pressurized to 80 mmHg. Data are mean ± SEM. **A.** AcH concentrations (μM) in the vessel bath during the time frame of the experimental procedure (30 min pretreatment followed by stimulation with increasing doses of PE), n = 6 for each concentration. **B.** PE-induced constriction in % of the o.d. in arteries pretreated with AcH for 30 min. Vehicle—n = 4; 0.1 μM AcH–n = 3; 1 μM AcH–n = 4; 5 μM AcH–n = 4, 10 μM AcH–n = 3. **C.** EC_50_ concentrations for the vasoconstrictor effects of PE (nM) from dose-response curves in B. *: p<0.05 vs. vehicle. **D.** PEth concentrations (μM) in the vessel bath during the time frame of the experimental procedure (30 min pretreatment followed by stimulation with increasing doses of PE), n = 6–9. **E.** PE-induced constriction in % of the o.d. in arteries pretreated with PEth for 30 min. Vehicle—n = 4; 0.25 μM PEth–n = 4; 1 μM PEth–n = 4; 2.1 μM PEth–n = 5. **F.** EC_50_ concentrations for the vasoconstrictor effects of PE (nM) from dose-response curves in D.

Next, we studied whether the ethanol metabolites acetaldehyde and phosphatidylethanol would influence PE-induced constriction of isolated arteries (Fig 2). We confirmed that vessel bath concentrations of both ethanol metabolites remained constant during the 30 min pre-incubation period and the subsequent period of stimulation with increasing doses of PE (Fig 2A and 2D). Addition of acetaldehyde or phosphatidylethanol alone to the vessel bath did not induce vasoconstriction. We observed that preincubation of arteries with acetaldehyde caused a left-shift of the dose-response curve for PE-induced vasoconstriction (Fig 2B). While acetaldehyde did not affect maximal PE-induced constriction and Hill-slopes of the PE-dose response curves, it significantly increased the potency of PE to induce constriction of isolated arteries 2.7-*fold* (Fig 2C, EC_50_ (nM, mean ± SEM)): vehicle– 977 ± 108; 5 μM acetaldehyde– 405 ± 42(p = 0.0084 vs. vehicle), 10 μM acetaldehyde– 362 ± 72 (p = 0.0087 vs. vehicle)). In contrast to ethanol and acetaldehyde, phosphatidylethanol did not affect the vasoconstrictor effects of PE (Fig 2E and 2F).

**Fig 3:**
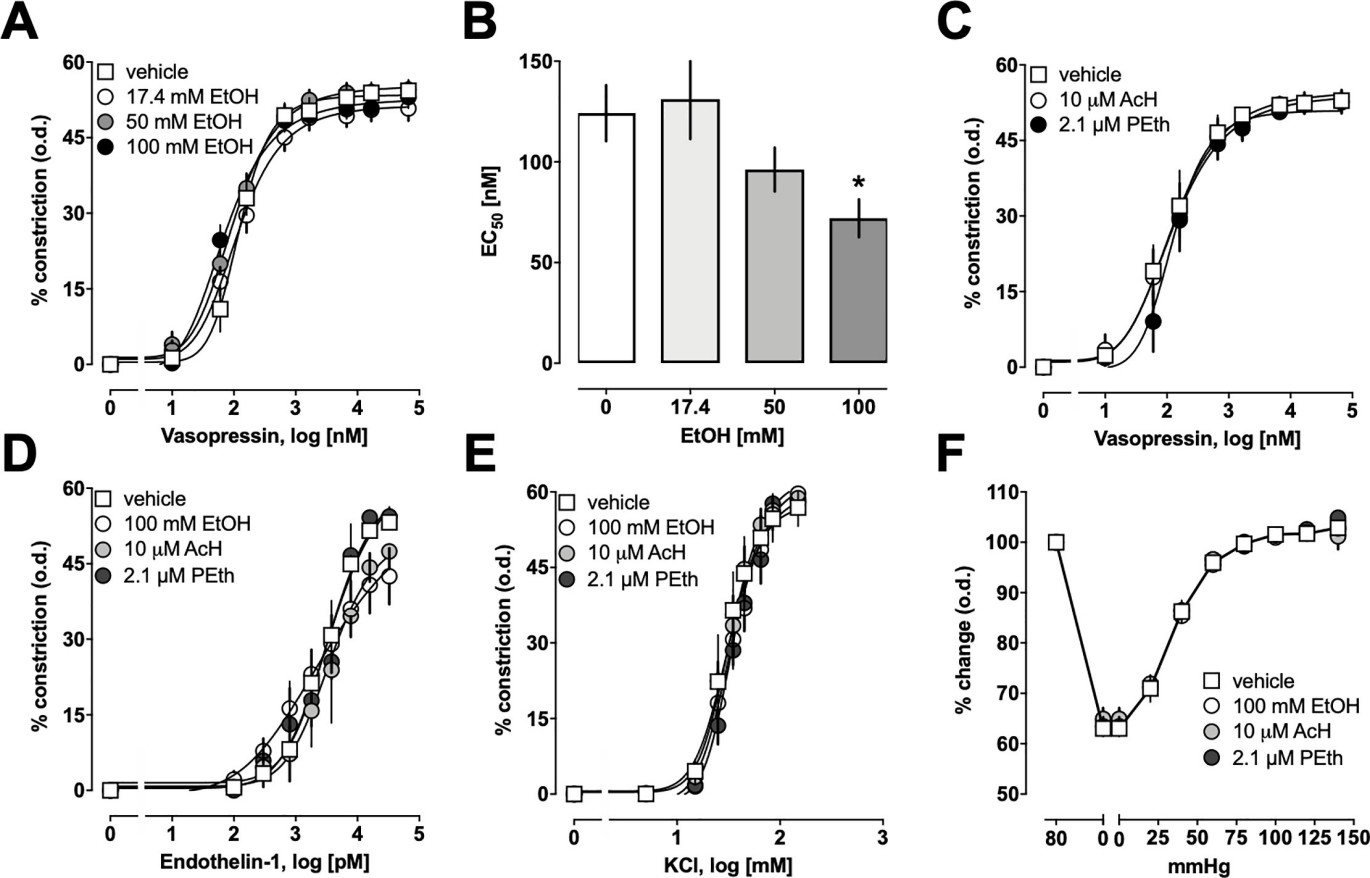
Effects of ethanol (EtOH), acetaldehyde (AcH) and phosphatidylethanol (PEth) on other vasoconstrictor effects and myogenic tone in mesenteric resistance arteries. Pressure myography experiments with arteries pressurized to 80 mmHg. Data are mean ± SEM. **A.** aVP-induced constriction in % of the outer diameter (o.d.) in arteries pretreated with single doses of EtOH for 30 min, as in Fig 1. Vehicle—n = 5; 17.4 mM EtOH–n = 5; 50 mM EtOH–n = 4; 100 mM EtOH–n = 5. **B.** EC_50_ concentrations for the vasoconstrictor effects of aVP (nM) from dose-response curves in A. *: p<0.05 vs. vehicle. **C.** aVP-induced constriction in % of the o.d. in arteries pretreated with AcH (10 μM, n = 6) or PEth (2.1 μM, n = 4) for 30 min. Vehicle–n = 6. **D.** Endothelin-1-induced constriction in % of the o.d. in arteries pretreated with EtOH (100 mM, n = 5), AcH (10 μM, n = 3) or PEth (2.1 μM, n = 3) for 30 min. Vehicle–n = 7. **E.** KCl-induced constriction in % of the o.d. in arteries pretreated with EtOH (100 mM, n = 3), AcH (10 μM, n = 3) or PEth (2.1 μM, n = 3) for 30 min. **F.** Effects of EtOH (100 mM, n = 3), AcH (10 μM, n = 3) and PEth (2.1 μM, n = 3) on myogenic tone. Vehicle–n = 3. Arteries were pretreated for 30 min. Data are expressed as % change of o.d. when arteries were exposed to various pressures (100% = o.d. at 80 mmHg).

The effects of ethanol on aVP-induced constriction of mesenteric resistance arteries are shown in Fig 3A. As in Fig 1A–1C, arteries were pre-incubated with ethanol for 30 min, followed by stimulation with aVP; ethanol concentrations in the vessel bath were not kept constant. With increasing concentrations of ethanol, the aVP dose-response curves shifted slightly to the left (Fig 3A). As observed for PE-induced vasoconstriction, maximal aVP-induced vasoconstriction and Hill-slopes of the aVP dose-response curves were not altered after pre-incubation of the arteries with ethanol. Ethanol, however, significantly reduced the EC_50_ concentration for the vasoconstrictor effects of aVP in a dose-dependent manner (Fig 3B). As compared with arteries that were not exposed to ethanol, pre-treatment of arteries with a starting concentration of 100 mM of ethanol increased the potency of aVP to induce vasoconstriction 1.7-*fold* (EC_50_ (nM, mean ± SEM)): vehicle– 124 ± 14; 17.4 mM ethanol– 131 ± 20; 50 mM– 96 ± 11; 100 mM– 72 ± 9 (p = 0.049 vs. vehicle)). In contrast to ethanol pre-treatment, pre-treatment of arteries with 10 μM of acetaldehyde or 2.1 μM of phosphatidylethanol did not affect the vasoconstrictor responses upon stimulation with aVP (Fig 3C). When tested at the highest concentrations that were used for PE- and aVP-dose response curves, ethanol, acetaldehyde and phosphatidylethanol did not affect constriction of mesenteric arteries upon stimulation with endothelin-1 (Fig 3D) or potassium chloride (Fig 3E). Furthermore, ethanol, acetaldehyde and phosphatidylethanol did not influence myogenic tone of the isolated arteries (Fig 3F).

## Discussion

In the present study we utilized pressure myography as a test platform to evaluate direct effects of ethanol and ethanol metabolites on the intrinsic function of mesenteric resistance arteries. Utilizing this test platform for detailed dose-response experiments, we provide evidence that ethanol selectively and dose-dependently sensitizes vasoconstriction upon activation of α_1_-adrenergic and arginine vasopressin receptors. Furthermore, we show that the ethanol metabolite acetaldehyde also sensitizes vasoconstriction upon activation of α_1_-adrenergic receptors but lacks effects on vasoconstriction upon activation of arginine vasopressin receptors. In contrast, the ethanol metabolite phosphatidylethanol did not affect intrinsic vasoconstrictor responses of isolated resistance arteries under our experimental conditions.

Acetaldehyde is produced through the enzymatic conversion of ethanol by alcohol dehydrogenase [28–33]. Acetaldehyde is a relatively short-lived metabolite which is further metabolized to acetate by acetaldehyde dehydrogenase [28, 30–34]. Multiple lines of evidence have previously suggested a role of acetaldehyde in the etiology of hypertension induced by ethanol consumption [35–41]. Phosphatidylethanol is the enzymatic product of the reaction of ethanol and phosphatidylcholine by phospholipase D [28, 42–46]. Phosphatidylethanol is of clinical interest as an alcohol consumption biomarker primarily due to its specificity and long biological half-life [28, 43–46]. The concentrations of ethanol and its metabolites that we used in the present study were selected to reflect clinically relevant blood ethanol concentrations and to include the ranges of blood acetaldehyde and phosphatidylethanol concentrations that have been observed in humans after ethanol consumption [46, 47].

Under our experimental conditions, ethanol evaporated from the vessel bath with a half-life of approximately 25 min. Thus, our findings indicate that a single and transient exposure of isolated arteries to ethanol is sufficient to sensitize intrinsic vascular function.

While ethanol dose-dependently sensitized vasoconstriction upon activation of the G protein-coupled α_1_-adrenergic and vasopressin receptors, ethanol did not influence vasoconstriction via activation of voltage-operated Ca^2+^ channels by KCl, vasoconstriction mediated through the G protein-coupled endothelin receptor or myogenic tone. Thus, our findings document selectivity of the observed effects of ethanol on intrinsic vascular function. Similarly, the findings that acetaldehyde also sensitized PE-induced vasoconstriction but did not affect vasoconstriction induced by other vasopressors, suggest selectivity of its sensitizing actions.

Although many pharmacological actions of ethanol are attributed to indirect effects related to its metabolites, its effects on metabolism and on the central nervous system, evidence suggests that ethanol can also directly bind to ethanol binding sites or pockets on proteins, through which it affects protein function [48, 49]. For example, ethanol binding has been reported for nicotinic acetylcholine receptors, N-methyl-d-aspartate receptors or G protein-gated inwardly rectifying potassium channels, and mutational analyses suggest that ethanol modulates γ-aminobutyric acid type A ρ1 receptor through binding to specific binding sites located in the 2^nd^ transmembrane domain of the receptor [48, 50–54]. Similarly, acetaldehyde is known to form stable and unstable adducts with proteins in vivo [55, 56]. The dose-effect profiles of ethanol and acetaldehyde that we observed in the present study indicate that their sensitizing effects on intrinsic vascular function are rapid, saturable and occur at clinically relevant concentrations, which could be consistent with direct binding of ethanol and acetaldehyde to the vasopressor receptors. The identification of possible receptor binding sites for ethanol and acetaldehyde, however, would require detailed structure-guided mutational analyses of possible binding sites. As such experiments are beyond the scope of the present study, the exact molecular mechanisms underlying the sensitizing effects of ethanol and acetaldehyde on intrinsic vascular function remain to be determined in the future.

It has been shown previously that acetaldehyde-induced hypertension in pithed rats can be attenuated with the α_1_-adrenergic receptor antagonists phentolamine and prazosin and by pre-treatment of animals with reserpine, a catecholamine-depleting sympatholytic [40]. These data have previously been interpreted to reflect acetaldehyde-induced release of catecholamines as a mechanism through which acetaldehyde induces vasoconstriction and hypertension in pithed rats [40]. The findings from the present study suggest that direct sensitizing effects of acetaldehyde on intrinsic vascular responsiveness to α_1_-adrenergic receptor activation likely contributed to these effects.

Previously, acetaldehyde concentrations of 1.9, 4.3 and 14 μM have been measured at blood ethanol concentrations of 17.1, 34.8 and 103.5 mM, respectively, one hour after ethanol gavage in rodents [57]. Thus, the dose-effect relationships for ethanol- and acetaldehyde-induced sensitization of α_1_-adrenergic receptor-mediated vasoconstriction that we observed in the present study match well with the relationship between ethanol and acetaldehyde concentrations in vivo [57]. Furthermore, 50% of east Asian populations exhibit an accumulation of acetaldehyde due to a variant of the ALDH2 gene, rs671 [29–34]. Our findings could provide a mechanism for the previously described relationship between increased acetaldehyde blood concentrations associated with the rs671 variant and an increased risk for the development of hypertension [30–34].

The effects of ex vivo exposure of mesenteric resistance arteries to ethanol on intrinsic vascular function that we observed in the present study resemble some but not all effects of ethanol consumption on intrinsic vascular function that have been observed previously [2, 6, 7, 21, 22, 58]. While differences between individual vascular beds, i.e. between mesenteric arteries, carotid arteries and the aorta, could account for such discrepancies, methodological differences in the assessment of intrinsic vascular function may also contribute. To the best of our knowledge, the majority of previous studies utilized wire myography to study the effects of ethanol consumption on intrinsic vascular function. While wire myography measures tension under isometric conditions and is prone to endothelial injury and non-physiological geometry and loading [59], pressure myography, which we employed in the present study, permits direct observation of vasoconstriction under isobaric conditions. Furthermore, our findings suggest that ethanol consumption resulting in blood ethanol concentrations above 17.4 mM leads to blood acetaldehyde concentrations that also sensitize intrinsic vascular function. Thus, it is conceivable that the parallel exposure of resistance arteries to ethanol, acetaldehyde and possibly other ethanol metabolites, such as fatty acid methyl esters, after ethanol consumption results in additional effects on intrinsic vascular function, beyond the effects of each molecule alone.

In the present study, however, we have limited our experiments to the study of the pharmacological effects of ethanol, acetaldehyde and phosphatidylethanol alone on intrinsic vascular function upon exposure to a single vasopressor. We abstained from combinatorial experiments to fully mimic in vivo conditions after ethanol consumption as such studies would not only require the exposure of mesenteric resistance arteries to various combinations of ethanol, acetaldehyde and other ethanol metabolites, but also to combinations of vasopressors. Such extensive studies, however, are technically not feasible within reasonable time frames and the interpretation of dose-response curves from such complex combination experiments appears daunting, if not impossible. Furthermore, we believe that the information gained from such experiments would only incrementally advance the key finding of the present study that ethanol and acetaldehyde have direct and selective effects on intrinsic vascular function.

Taken together, our findings demonstrate that ethanol and acetaldehyde selectively sensitize intrinsic constrictor responses of mesenteric resistance arteries upon activation of vascular α_1_-adrenergic and/or vasopressin receptors at clinically relevant concentrations. While the precise molecular mechanisms underlying their pharmacological effects remain to be determined, our findings further support the concept that enhanced vasoreactivity to endogenous vasoactive hormones contributes to the development of hypertension induced by ethanol consumption. Ex vivo exposure of mesenteric resistance arteries in pressure myography experiments resembles important aspects of vascular alterations that have been observed after ethanol consumption, and thus could serve as a simple test platform to evaluate therapeutic interventions that are aimed to mitigate vascular effects of ethanol and acetaldehyde.

## Supporting information

S1 Datasets All data sets are provided in this file. (XLSX)
